# Application of Ultrasound Treatments in the Processing and Production of High-Quality and Safe-to-Drink Kiwi Juice

**DOI:** 10.3390/foods13020328

**Published:** 2024-01-20

**Authors:** Sharayu Bhutkar, Teresa R. S. Brandão, Cristina L. M. Silva, Fátima A. Miller

**Affiliations:** CBQF—Centro de Biotecnologia e Química Fina—Laboratório Associado, Escola Superior de Biotecnologia, Universidade Católica Portuguesa, Rua Diogo Botelho 1327, 4169-005 Porto, Portugal; sharayu.bhutkar16@gmail.com (S.B.); tbrandao@ucp.pt (T.R.S.B.); clsilva@ucp.pt (C.L.M.S.)

**Keywords:** chlorophylls, inactivation kinetics, *L. innocua*, minerals, phenolics, thermal treatment, thermosonication

## Abstract

This study explores the potential of thermosonication as an alternative to traditional heat treatments, such as pasteurization, in the processing of fruit juices. Conventional methods often lead to undesirable quality changes in fruit juices, whereas thermosonication offers promising results regarding microbial inactivation and quality preservation. This work focused on the inactivation kinetics of *Listeria innocua* 2030c, a surrogate for pathogenic *L. monocytogenes*, in kiwifruit juice using thermosonication at 45 °C, 50 °C, and 55 °C. These treatments were compared with equivalent heat treatments. Quality attributes of the juice were also evaluated to assess process efficiency. Survival data of *L. innocua* were fitted with the Weibull model, estimating first decimal reduction times (δ) and shape parameters (n). The results reveal temperature and process dependencies on δ, while n remains mostly temperature and treatment independent. Thermosonication outperforms heat treatment, achieving higher *L. innocua* reductions while retaining quality attributes like pH, soluble solid content, and total phenolics and chlorophylls. Thermosonication at 55 °C stands out, providing a 6.2-log-cycle reduction in just 3 min with superior quality retention. These findings highlight the synergistic effect of temperature and ultrasound, making mild heat processes feasible while enhancing product quality. Thermosonication, particularly at 55 °C, emerges as an effective alternative to traditional thermal treatments for fruit juices, offering improved microbial safety without compromising product quality.

## 1. Introduction

The demand for natural, healthy, and convenient beverages has risen significantly in recent years. Consumers are increasingly seeking alternatives to traditional fruit juices that offer superior nutritional value and meet stringent safety standards. Kiwi, recognized for its exceptional taste and rich nutritional profile, has gained popularity as a potential fruit for juice production. However, the processing and production of kiwi juice present several challenges due to the fruit’s susceptibility to chemical, physical, and microbiological changes. Therefore, producing microbiologically safe, healthy, highly nutritious juices with natural characteristics is the overall goal.

During the last few years, illness outbreaks associated with fruit juices have been a growing public health concern because of the rise of fresh juice consumption due to consumers’ demands for convenient, healthy, and minimally processed food. Most fresh juices contain a microbial population ranging from 4 to 6 log units per gram [1]. Therefore, Food and Drug Administration (FDA) demands that fruit juice industries use treatments to achieve a 5-log microbial reduction [2].

Conventional heat processes such as pasteurization are currently applied in the food industries to inactivate enzymes and microorganisms. Enzymes relevant to kiwi juice quality are mentioned to be present in small amounts and unlikely to be stable at low pH levels [3]. This could include pectin methylesterase (PME), which is involved in softening, and polyphenol oxidase (PPO), which can contribute to the browning process. However, as confirmed by several researchers [3,4], PME has a natural inhibitor—a proteinaceous PME inhibitor (PMEI) that has been shown to inhibit the PME to obtain cloud-stable juices. Unlike other fruit juices, kiwi juice does not oxidize since it has low PPO activity and high levels of ascorbic acid. However, thermal treatment frequently leads to unwanted changes in fruit juices’ physical, chemical, biological, and organoleptic properties, such as color alterations, loss of nutrients, and off-flavor development [5]. Therefore, the demand for new technologies that minimize the harmful effects of heat treatment on the nutritional content and overall quality content of juices is increasing. Moreover, as consumers become more aware of the correlation between food, diet, and health, and interested in natural ingredients, the development of mild processing technologies is growing [6].

Ultrasound technology has emerged as a promising solution to address these challenges and develop the production of high-quality and safe-to-drink juices. Using ultrasound coupled with mild thermal treatments (i.e., thermosonication) in fruit juice processing has shown remarkable potential in decreasing processing time, reducing energy consumption, increasing efficiency, enhancing product quality, extending shelf life, and ensuring microbiological safety [7]. This is achieved due to the phenomena of cavitation that is induced during ultrasound processing. Cavitation refers to microbubbles’ formation, growth, and implosive collapse within a liquid medium [8]. The collapsing bubbles cause microbial cell disruption by generating high-temperature hot zones and pressure in the medium [9].

According to the literature, many works have been published on ultrasound/thermosonication applied to fruit juices, especially on orange [10,11,12], apple [13,14], pineapple [15,16], strawberry [17], and guava [18] juices. All agreed that this process effectively enhances the juice quality parameters and allows for better microbial inactivation results than heat treatments. However, studies on the impact of ultrasonication on kiwi juice are scarce. Only two works have been conducted to evaluate the effect of ultrasounds on kiwi juice quality [19,20]. They reported an improvement in bioactive compounds such as total phenolics, flavonoids, and antioxidant activity and a significant decrease in ascorbic acid and total proteins [19]. Similarly, an enhancement of the rheological properties, color attributes, cloudiness, and water-soluble pectin of kiwifruit juice was also described [20]. To the best of the authors’ knowledge, no works investigating ultrasonication applied to kiwi juice for microbial inactivation have been published.

The main objective of this study was to assess the impact of thermosonication, at different temperatures, on the kinetic survival behavior of *L. innocua* (a surrogate for the pathogenic *L. monocytogenes*) in kiwifruit juices. The decimal reduction times and survival curves shape parameters were quantified by fitting the data with a Weibull model. Additionally, the effects of thermosonication on several quality attributes of the juices were compared with those obtained through traditional heat treatments using the same temperatures. Those characteristics encompass physicochemical features such as color, soluble solid content, pH, cloud value, bioactive compounds (total phenolics and chlorophylls), and minerals. By addressing these objectives, the study aimed to provide insights into the efficacy of thermosonication as an alternative processing technique and its impact on microbial safety and the quality attributes of kiwifruit juices.

## 2. Materials and Methods

### 2.1. Juice Preparation

Kiwifruit (*Actinidia deliciosa* cv Hayward) was acquired at a local supermarket in Porto, Portugal, at the commercial maturity stage and stored overnight at 4 °C. Three kiwifruits were selected randomly for each experiment. Using a peeler, the kiwifruit peel was removed manually, and the remaining kiwifruit was sliced into small pieces. The juice was extracted using a household centrifuge with a power of 1500 W at room temperature (Centrifugal Juicer Excel JE850, Kenwood, London, UK).

The pH of the kiwifruit juice was modified to 3.6 by incorporating natural cantaloupe melon (*Cucumis melo* L. var. *reticulatus*) juice. The pH was measured by continuously agitating the kiwifruit juice and gradually adding melon juice until the desired pH was reached. This was done because the *Listeria* strain is susceptible to the naturally low pH of kiwifruit juice.

### 2.2. Treatments

#### 2.2.1. Thermosonication

Juice samples (20 mL) were placed in 50 mL glass beakers closed with parafilm and submitted to thermosonication treatment. Thermosonication was carried out using an ultrasonic probe (Model CL-334, Qsonica, Newtown, Connecticut, USA) at a constant frequency of 20 kHz and discontinued pulsation (10 s on, 5 s off). The ultrasonic tip (diameter 13 mm) was inserted 1 cm inside the juice sample, and the amplitude was set at 80%. The ultrasonic processor was coupled with a water bath and ice water to maintain the juice at the desired and constant temperature of 45 °C, 50 °C, and 55 °C. In addition, the ultrasonic processor has a temperature probe connected to the system to monitor the juice samples’ accurate temperature. For microbiological analysis, sampling was conducted every 1 min for 15 min, every 1 min for 10 min, and every 0.5 min for 3 min at 45 °C, 50 °C, and 55 °C, respectively. After treatment, the samples were rapidly placed in ice water to cool them to room temperature. The processes were replicated three times.

#### 2.2.2. Thermal Treatment

Thermal treatments at the same temperatures were applied to freshly squeezed juices to use as the control, and sampling was carried out for microbiological analysis every 10 min for 60 min, every 2.5 min for 25 min, and every 2.5 min for 15 min at 45 °C, 50 °C, and 55 °C, respectively. The processes were replicated three times.

Quality analyses (physicochemical characteristics, bioactive compounds, and minerals) were conducted in triplicate before (in freshly squeezed kiwi juices at an ambient temperature of around 18 °C) and at the end of each thermosonication and thermal treatment.

### 2.3. Physicochemical Analysis

#### 2.3.1. pH, Soluble Solid Content, and Cloud Value

pH values were attained using a pH meter (GLP 22, Crison Instruments, Barcelona, Spain). Soluble solid content (SSC, °Brix) was evaluated using a Palette PR-32 digital refractometer (Atago, Tokyo, Japan). To determine the cloud value, 10 mL of juice was centrifuged at 5000 rpm for 10 min at 4 °C. The supernatant absorbance was measured at 660 nm using a UV/VIS spectrophotometer (Model 5625, ATI Unicam, UK), and distilled water was used as a blank [20].

#### 2.3.2. Color

The color of juice samples was determined using a Minolta CR-400 colorimeter (Konica-Minolta, Osaka, Japan), where the CIE L*a*b* system was applied. Before each sample color measurement, the colorimeter was adjusted using a standard white plate for accuracy. The measurements were expressed in L*, a*, and b* values, with L* as the brightness coordinate determining a color’s whiteness value from 0 (black) to 100 (white). The green–red chromaticity value a* represents the spectrum from −60 (green) to +60 (red), and the chromaticity value b* for the blue–yellow spectrum ranges from −60 (blue) to +60 (yellow) [21].

The total color difference (TCD) was used to evaluate the color changes between untreated and treated samples and was calculated using the following formula:

(1) $$TCD=\sqrt{{\left(L_{0}^{*}-L^{*}\right)}^{2}+{\left(a_{0}^{*}-a^{*}\right)}^{2}+{\left(b_{0}^{*}-b^{*}\right)}^{2}}$$

where L_0_*, a_0_*, b_0_* represent values of fresh juice samples, and L*, a*, b* represent values of treated juice samples.

The *chroma* and *hue angle* were also calculated according to Equations (2) and (3), respectively:

(2) $$Chroma=\sqrt{{a^{*}}^{2}+{b^{*}}^{2}}$$

(3) $$Hue\;angle={tan}^{-1}\left(\frac{b^{*}}{a^{*}}\right)+180,\;when\;a^{*}<0$$

### 2.4. Total Phenolics and Chlorophylls

The phenolic contents in kiwi juice samples were determined using the Folin–Ciocalteu reagent by blending 25.0 mL of juice with 50.0 mL of 100% methanol using an Ultra-Turrax^®^ homogenizer (Ika digital T25, IKA-Werke GmbH & Co.KG, Staufen, Germany). The mixture was centrifuged at 5000× *g* for 10 min at 4 °C. The method followed the procedure outlined by Fundo et al., (2018) [22], where standard solutions were created with varying concentrations of gallic acid. The reaction occurred by combining the standard solution or juice sample with Folin–Ciocalteu’s phenol reagent, Na_2_CO_3_ 75 g L^−1^, and distilled water. After incubating in the dark at room temperature for 1 h, absorbance was measured at 750 nm using a UV/VIS spectrophotometer (Model 5625, ATI Unicam, UK). Data were presented in μg of gallic acid equivalents (GAE) per mL of juice.

Chlorophyll a and b were also extracted by blending 7.0 g of kiwi juice with 50.0 mL of 100% methanol with the Ultra-Turrax. The mixture was centrifuged at 5000× *g* for 10 min at 4 °C and filtered with filter paper. Chlorophyll a and b were determined via spectroscopy [23], and the total chlorophyll content was estimated from the sum of chlorophyll a and chlorophyll b. Data were expressed as μg/mL of juice.

### 2.5. Minerals

The assessment of mineral content in the kiwi juices was conducted according to the method outlined by Chatelain et al. (2014) [24], using a Berghof microwave digestion system. First, the juice samples were digested in an acid solution using a Berghof microwave digestor (Speedwave four DAP-100+, Berghof, Germany). Spontaneous reactions were avoided by heating in steps. Stabilized 65% HNO_3_ and 30% H_2_O_2_ were oxidant agents for sample digestion. The digestion procedure was conducted in five steps: 1–170 °C/10 min, 2–200 °C/15 min, 3–190 °C/10 min, 4–100 °C/2 min, and 5–100 °C/2 min. Lastly, the digested sample was diluted to a final volume of 15 mL with distilled water and analyzed for minerals using the ICP-OES technique. The concentrations of phosphorus (P), magnesium (Mg), calcium (Ca), sodium (Na), and potassium (K) were assessed using an inductively coupled plasma–optical emission spectrometer (PerkinElmer^®^, 7000 DV, Waltham, MA, USA). The results were reported in mg/mL.

### 2.6. Microbiological Analysis

*L. innocua* 2030c, supplied by Public Health Laboratory Service—PHLS (Colindale, UK) private collection—was used as a surrogate of *L. monocytogenes*. Subcultures were made according to Miller et al.’s (2009) [25] methodology until a concentration of 10^9^ CFU/mL of the bacterial strain was achieved. The juice samples were artificially inoculated by adding 1 mL of the subculture to 19 mL of juice, obtaining a final contamination of approximately 10^7^ CFU/mL.

The *L. innocua* was quantified in triplicate, before and after each treatment, through decimal dilutions and was spread on PALCAM agar containing selective supplement (BIOKAR Diagnostics, Pantin, France). Plates were incubated at 37 °C for 48 h. The viable *L. innocua* colonies were enumerated using the Plate Count Method and expressed as CFU/mL of juice.

### 2.7. Modeling of L. innocua Inactivation

The Weibull model was used to describe the log-survival data of the *L. innocua* obtained in the thermal and thermosonication treatments [14]:

(4) $$log\left(\frac{N}{N_{0}}\right)=-{\left(\frac{t}{\delta }\right)}^{n}$$

where N is the microbial load after treatment (CFU/mL), N_0_ is the juice initial microbial load (CFU/mL), t is the treatment time (min), δ is the first decimal reduction time, i.e., the time required to achieve the first 1-log reduction (min), and n is a shape parameter (dimensionless). A shape parameter n < 1 indicates concavity upward, n > 1 indicates concavity downward, and n = 1 demonstrates linearity. Equation (2) was fitted to the log-survival data of *L. innocua* for all treatments. Non-linear regression analysis was conducted using IBM SPSS Statistics 27 for Windows^®^ (SPSS Inc., Armonk, NY, USA).

The goodness of the regressions was evaluated by checking the residuals’ randomness and normality (mean zero and constant variance). The Kolmogorov–Smirnov test was used to test normality, and the Breusch–Pagan test based on X^2^ was used to test the constancy of variance (homoscedasticity). The significance level was set to 5% in all tests performed. The adjusted coefficient of determination (R^2^_adjusted_) and the root mean squared error (RMSE) between all experimental and predicted data were also calculated. The accuracy of the model parameters was determined by calculating confidence intervals at 95%.

### 2.8. Statistical Analysis

One-way ANOVA was utilized to recognize variations among all treatments applied to kiwi juice, and all characteristics analyzed. Tukey’s and Duncan’s tests were performed for post hoc differences of means. The data’s normal distribution and uniform variance were examined through the Shapiro–Wilk test for normality and Levene’s test for homoscedasticity, respectively. The significance level assumed was 5% (*p* < 0.05 means a statistically significant test result). Results were presented as the mean value along with a 95% confidence interval.

A Principal Component Analysis (PCA) with varimax rotation was performed to reduce the number of variables based on their correlations to obtain principal components (clusters of variables). The criteria for setting the number of relevant principal components were the scree plot, eigenvalues higher than 1, and the proportion of variance explained by each component. A threshold of 59% of the cumulative variance of the components was set for such a decision. The criterion for variable inclusion in the principal components was loadings above 0.6 in the component’s matrix.

Data analyses were determined using IBM SPSS Statistics 27 for Windows^®^ (SPSS Inc., Armonk, NY, USA).

## 3. Results and Discussion

Due to some variability between fresh samples, and since the work aimed to observe the impact of each treatment on the juice quality parameters, data were normalized according to the values obtained for untreated (fresh) juice. The results of the physicochemical, phytochemical, and mineral compositions of fresh kiwi juice are presented in Table 1.

### 3.1. Treatment Effects on Physicochemical Analysis

**Figure 1:**
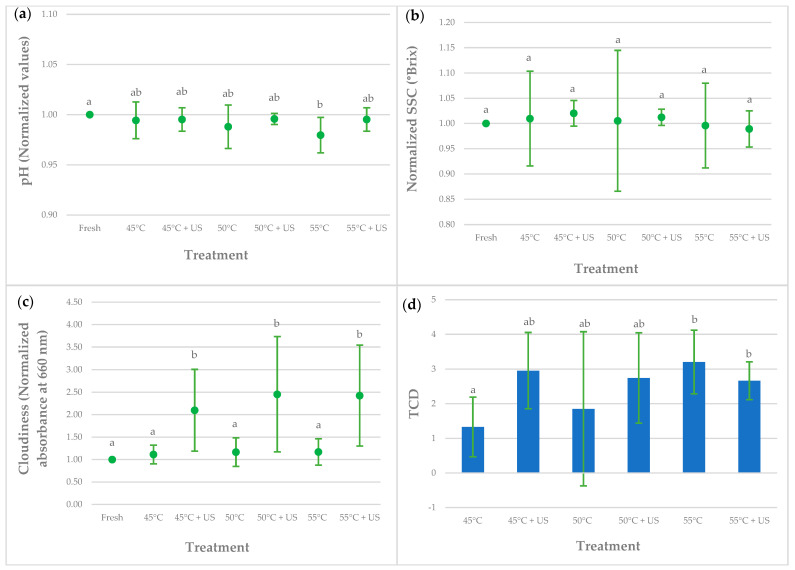
Effect of thermosonication and thermal treatments on the pH (**a**), SSC (**b**), cloud value (**c**), and total color difference (**d**) of kiwi juice samples. The presented data represent mean values, with the bar limits indicating 95% confidence intervals. For a given treatment, values with different letters differ significantly (*p* < 0.05).

The results for pH, SSC, cloud value, and color in fresh (untreated), thermally treated, and thermosonicated kiwi juices are presented in Figure 1.

pH is the most influential among the several intrinsic parameters that affect the quality and safety of juices. Since acidic or alkaline conditions inhibit or promote microbial growth, pH is primarily used in food matrixes to indicate safety. Variations in pH can impact the flavor, consistency, and shelf life of fruit juices. Fruit juices usually have low pH values between 2 and 5 due to organic acids [26]. To maintain consistency in the quality of juice, monitoring the pH is essential. The results indicate that the applied processes had no significant impact on all the kiwi juice samples, except for the heat-treated at 55 °C, where the pH was significantly lower than the untreated juice but equal to the other treated juices (Figure 1a). This decrease can probably be attributed to some chemical or enzymatic reactions that occur at high temperatures. For instance, the heat treatment may lead to the degradation of ascorbic acid, resulting in the release of hydrogen ions and thus lowering the pH. Other researchers also reported similar findings, stating no significant changes in the pH values of untreated and thermosonicated fruit juices [27,28].

SSC is typically dominated by reducing and non-reducing sugars, with minor contributions from organic and amino acids and soluble proteins [29]. It is therefore considered an essential criterion for determining fruit flavor and ripeness. The SSC value of fresh juice showed some variability (Table 1), probably due to different internal fruit maturation. As shown in Figure 1b, SSC values remained significantly unaffected by any of the treatments. Although all the SSC values were very similar, it was possible to follow that the confidence intervals for the heat-treated samples were very high compared with the thermosonicated ones. These values showed that although all the treatments allowed total retention of SSC in kiwi juice, considerable variability was attained for the heat-treated samples. This variability may be influenced by the different effects that thermal and thermosonication processes have on the chemical and physical properties of kiwi juice.

Fruit juices are often sold as clear or cloudy products with varying densities. Both clear and cloudy varieties may have market demand. In fruit juices, cloudiness is a desired quality parameter. It relates to cellulose particles, hemicelluloses, protein, lipids, pectin, and other minor components [30], enhancing the fruit juices’ colors and flavors. From the results of Figure 1c, it can be concluded that thermosonication was more successful than heat treatment in improving the cloud values of kiwi juices. This is due to the phenomenon of cavitation that causes a breakdown of the juice’s large solid particles into smaller molecules due to mechanical forces and facilitates homogenization. Seshadri et al. [31] suggested that ultrasound may break linear pectin molecules, and Krešić et al. [32] proposed changes in protein conformation and structure. These findings are in agreement with the results of Wang et al. [20], where the cloud value of kiwifruit juice improved significantly after ultrasound processing compared to untreated samples. Other researchers support this conclusion concerning other fruit juices, namely in apple [33], orange [34], and peach [35] juices.

Although not significantly different, the cloud values of thermosonicated samples changed with temperature, meaning that kiwifruit juice’s cloudiness depends on the process temperature and the ultrasound processing time. Indeed, Wang et al. [20] reported that the cloudiness of kiwi juice increased during the first 8 min of ultrasound processing, reached the maximum threshold, and then decreased with a further increase in processing duration. Cervantes-Elizarrarás et al. [36] also reported a rise in the cloud index of fruit juices with the treatment temperature.

Color is a critical parameter affecting food consumer acceptance [37]. Hence, total color difference (TCD) analysis is required to detect if a treatment altered the food product’s quality. TCD values of thermally treated and thermosonicated kiwi juices are depicted in Figure 1d. As can be observed, the juice color suffered alterations after heat treatments at 45 °C (TCD = 1.33 ± 0.86) and 50 °C (TCD = 1.85 ± 2.22) and also after thermosonication at 45 °C (TCD = 2.95 ± 1.10) and 50 °C (TCD = 2.74 ± 1.30). However, the color alterations of these juices are not significantly different between these treatments. According to Dr. Lange’s scale [38], the mean value of total color difference regarding the untreated sample can be considered “distinct”. By comparing the treatments, color was better preserved at 45 °C, since it had the lowest TCD value. And since the mean TCD value was between 0.5 and 1.5, the color difference is considered “small”. This value was significantly different from the ones attained for thermosonication at 55 °C (TCD = 2.67 ± 0.54) and heat treatment at 55 °C (TCD = 3.20 ± 0.92). The fresh and treated juice color at 55 °C presented a very “distinct difference”. The significant difference between them could be attributed to the higher temperature applied.

Since some differences in one of the three color coordinates might not be detected via TCD calculation, the color parameters were analyzed separately. Therefore, Table 2 presents brightness (L*), greenness (a*), and yellowness (b*) for thermally treated and thermosonicated kiwi juice.

The results indicate that lightness increased compared to the fresh juice for both thermosonication and thermal treatments. Indeed, except for heat treatments at 45 °C and 50 °C, the other processes significantly increased the L* value. Data obtained for a* and b* values revealed that these parameters seem to rise with the temperature increment for heat-treated juices. On the other hand, in thermosonicated kiwi juices, these parameters decreased with higher temperatures. This was also in line with Tiwari et al.’s [34] research, where orange juice subjected to sonication exhibited the same color pattern degradation. The higher L value of the juice indicates it is becoming more transparent as previously formed colored compounds are being destroyed. The decreases in a* and b* can be linked to the increased degree of browning [39]. Nevertheless, all these values are similar to that of the fresh sample. The chroma and hue angle were calculated to exclude the influence of the proposed treatments on other important color parameters. Data are included in Table S1. Chroma values ranged between 4.93 ± 0.86 for thermosonication and 8.05 ± 1.51 for heat treatment, both at 55 °C. Although all the values were significantly similar to the fresh sample (6.58 ± 0.53), the heat treatment increased color intensity, while ultrasonication decreased color strength. Regarding hue angle, except for heat treatment at 45 °C (109.39 ± 3.24), this parameter suggests a shift toward a slightly more yellowish-green or yellow tone. This change might imply a warmth of the color of the treated samples (117.05 ± 3.68) compared with the fresh one (112.56 ± 1.30).

### 3.2. Treatment Effects on Total Phenolics and Chlorophylls

**Figure 2:**
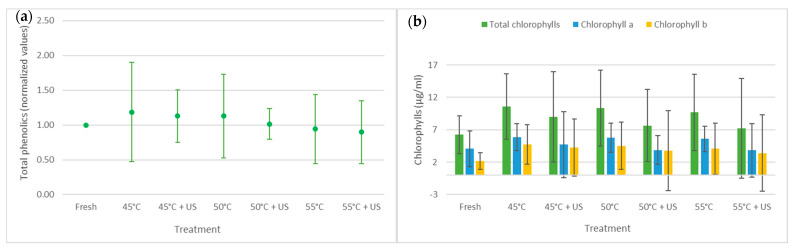
Impact of thermosonication and thermal treatments on total phenolics (**a**), total chlorophylls and chlorophyll a and b (**b**) of kiwi juice samples. Data represent mean values, with the bar limits indicating 95% confidence intervals.

The results for total phenolics and chlorophylls in fresh (untreated), thermally treated, and thermosonicated kiwi juices are presented in Figure 2.

Total phenolics did not show significant changes as a result of any of the treatments when compared to fresh kiwi juice. Also, no significant differences between thermosonication and thermal treatments were observed. However, the total phenolics obtained after treatments at 45 °C, 50 °C, and thermosonication at 45 °C slightly improved their content, probably due to a higher treatment time. This rise in total phenolics may be linked with their release due to cell wall disintegration via cavitation during thermosonication. According to Wang et al. [19], ultrasound treatment for 16 min showed improved and the highest total phenolics content in kiwi juice, compared to fresh or treated samples for 4, 8, and 12 min. Nayak et al. [40] also noticed a gradual increase in total phenolics as the processing time and temperature increased. In the present work, an increase in the treatment temperature implies a decrease in the processing time, which reduces the total phenolics.

Typically, phenolic compounds are found in plant tissues in soluble form. High-intensity ultrasound processing may cause cell walls and vacuoles in plant tissues to rupture, thus increasing the juice total phenolics [41]. These increases may also be attributed to the improvements in the extraction efficiency of the thermosonication process and the release of bound forms of phenolic acids due to the cavitation effect [40].

Total chlorophylls were not significantly affected by any of the treatments when compared to fresh kiwi juice. Likewise, no significant differences were detected between thermosonication and thermal treatments. Chlorophyll a and b seem to increase after treatments, but their contents were not significantly different in all situations. However, in some cases, this result lacks precision, as large meaningless confidence intervals at 95% were attained. Nevertheless, higher values were found for chlorophyll a than chlorophyll b, which is usually reported in the literature.

Zhang et al. [42] noticed increased total chlorophyll content when thermosonication was applied to daylilies. Furthermore, the chlorophyll amount revealed no significant differences between heat-treated and thermosonicated watercress samples [43]. It is widely accepted that chlorophylls are susceptible to heat [44]. Therefore, chlorophyll content decreased with the increase in the treatment temperature. Some studies also confirmed that ultrasound is an efficient process for chlorophylls’ higher extraction yield [45,46]. However, when ultrasounds are coupled with heat treatment, many variables impact the final content of chlorophylls. Ahmed et al. [47] reported a significant increase in the total chlorophyll contents of wheat plantlet juice during ultrasounds at 30 °C and 45 °C, while a reduction was observed when the ultrasound temperature reached 60 °C.

### 3.3. Treatment Effects on Minerals

Each mineral is vital in a variety of human bodily processes. They are considered essential nutrients since the body does not produce them and should be acquired from food or supplements to satisfy daily requirements [48]. Fruits are known as excellent sources of minerals. Treatment effects on minerals are shown in Table 3.

The results revealed that for Ca, thermosonication and thermal treatments had no significant effects compared to fresh kiwi juice. Regarding P, it can be noted that thermosonicated samples at all temperatures were equivalent to fresh juice; significantly higher values were observed in heat-treated samples at 50 °C and 55 °C. In the case of Mg, its concentration increased after all treatments except for ultrasonication at 45 °C. Only ultrasonicated juices at 55 °C presented a lower content for Na. This agrees with the work on thermosonicated black grape juice, which also observed a statistically significant decrease in Na contents as the thermosonication process was applied [49]. Heat-treated juice at 45 °C and thermosonicated at 50 °C had significantly lower K contents than fresh juice.

For apple juice, Abid et al. [50] reported contradictory results for P, Na, K, Ca, and Mg. Regarding the yolk of ultrasound-treated eggs, the results for minerals P and Mg are in agreement with the studies of Sert et al. [51]. However, opposite tendencies were observed for the other minerals. These findings suggest that the impact of the process on these minerals depends on the specific mineral and food matrix.

The estimated average requirements for K, Na, P, Ca, and Mg are 3500, 500, 1250, 1000, and 300 mg/day, respectively. Based on the findings, a 200 mL glass of thermosonicated kiwi juice at the highest temperature not only meets the recommended potassium dose but also serves as a significant source of other essential minerals.

### 3.4. Inactivation of L. innocua

**Figure 3:**
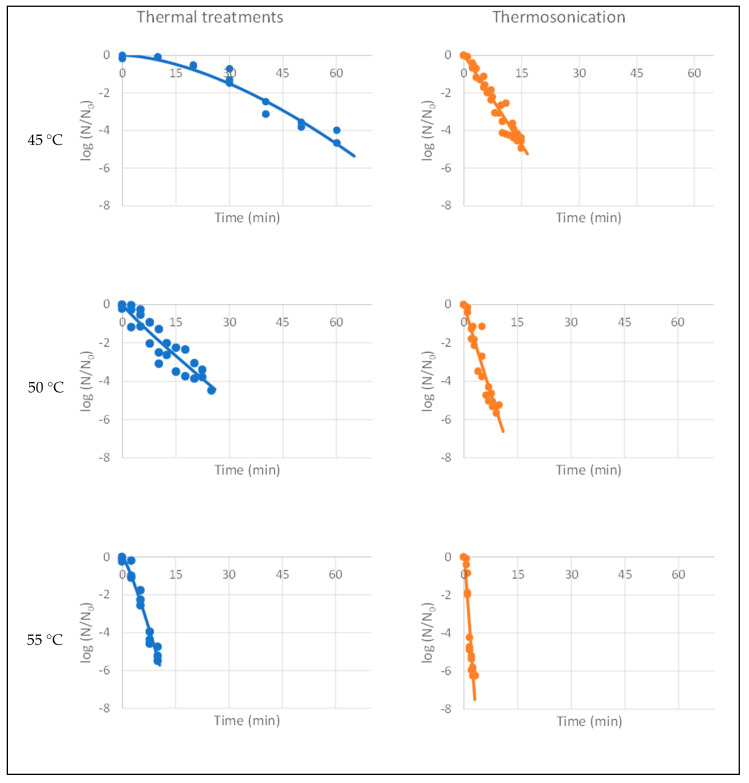
Log-survival data of *L. innocua* in kiwi juice submitted to thermal and thermosonication treatments. Lines represent the Weibull model fits (Equation (2)).

To determine the treatment time required to achieve the FDA-recommended 5-log reduction, the survival of *L. innocua* in kiwi juices was studied over time under thermal and thermosonication treatments. The Weibull model (Equation (2)) was adequately fitted to the inactivation experimental data of *L. innocua*, and kinetic parameters were estimated for the analyzed conditions. The residuals were randomly and normally distributed in all cases, showing homoscedasticity (*p* > 0.05). Table 4 includes the model parameters and their confidence intervals at 95% for all treatments, as well as the values of R^2^_adj_ and RMSE. The *Listeria* kinetic behavior for all treatments is visualized in Figure 3.

The values of R^2^_adj_ were very high for almost all cases, varying from 0.90 to 0.95. However, for heat treatment at 50 °C, a noticeable dispersion of experimental data was observed, resulting in an adjusted coefficient of determination of 0.85. Regarding the RSME, the lower the value, the better the model’s performance. This is consistent with the R^2^_adj_, where the lower temperatures presented the best model fits.

The parameter δ represents the first decimal reduction time, which is the time needed to attain the first 1-log reduction. Table 4 shows that, for the same temperatures of 45, 50, and 55 °C, these values are 86%, 71%, and 82% higher for heat treatments compared to thermosonicated treatments. These results indicate that *L. innocua* inactivation occurs more rapidly (indicating greater sensitivity) in thermosonicated treatments, thus demonstrating the efficiency of the process. Another conclusion is that, under the same treatment, the value of δ decreases with increasing temperature. Several researchers reported that the rise in temperature improves microbial inactivation in thermosonication treatments as it enhances the lethal process effect [52].

Regarding the shape parameter n, it is interesting to observe that except for heat treatment at 45 °C, all the others were of the same magnitude, revealing identical curve shapes. For heat treatment at 45 °C, the n parameter was 1.64 ± 0.30. In this case, the curve concavity was downward. Although exhibiting some deviations, the other treatments showed n values very close to 1, indicating that the curves are practically linear (Figure 3).

As depicted in Figure 3, thermosonication at all the tested temperatures demonstrated a complete 5-log-cycle reduction, aligning with FDA requirements for efficient juice processing. The same did not occur for heat treatments at 45 °C and 50 °C, as they only achieved 4.5-log-cycle reductions in *L. innocua*. As can be observed, processing times are significantly lower for thermosonication: 15, 10, and 3 min for 45 °C, 50 °C, and 55 °C, respectively. Applying heat treatments takes 60, 25, and 10 min to achieve 4.6-, 4.5-, and 5.5-log-cycle reduction at 45 °C, 50 °C, and 55 °C, respectively. These results demonstrate that combining ultrasound with moderate heat produces a synergistic effect, suggesting this process as an effective treatment for inactivating microorganisms in food and beverages.

According to a study by Kiang et al. [53], the ultrasound treatment of mango juice at 60 °C for 7 min at 25 kHz led to a 5-log reduction in *E. coli* O157H:7. *Staphylococcus aureus* was reduced by 5.5 logs when orange juice was treated at 55 °C for 30 min at 30 kHz [52]. In this situation, the microbial inactivation mechanisms can be explained by (i) the rise in temperature leading to a decline in juice viscosity, enhancing the production of cavitation bubbles and resulting in more effective bubble collapse, and (ii) higher temperatures weakening the external membrane of bacterial cells, making the cells more susceptible to ultrasound [54].

Most researchers consider that the antimicrobial effect of thermosonication is related to pressure changes that induce the cavitation phenomena. Ultrasonic waves create bubbles that quickly collapse, leading to cell breakdown, cell membrane destruction, and DNA damage, all contributing to microorganisms’ disruption [7]. It is also of utmost importance to point out that the efficiency of thermosonication in inactivating microorganisms relies on various parameters, including temperature, treatment time, type of microorganism, product pH, ultrasound frequency, amplitude, and reactor.

The kinetic behavior of *L. innocua* at 45 °C exhibited a sigmoidal tendency characterized by an initial shoulder succeeded by a period of maximum inactivation rate. The presence of a shoulder can be explained on the basis that microbial viability loss is not solely linked to one critical target; instead, several critical components per cell must be damaged to induce inactivation [55].

Thermosonication at 55 °C is the optimum treatment for inactivating *L. innocua* in kiwi juice, as it has the lowest δ value and achieves a 6.2-log-cycle reduction in just 3 min.

### 3.5. Principal Component Analysis

**Figure 4:**
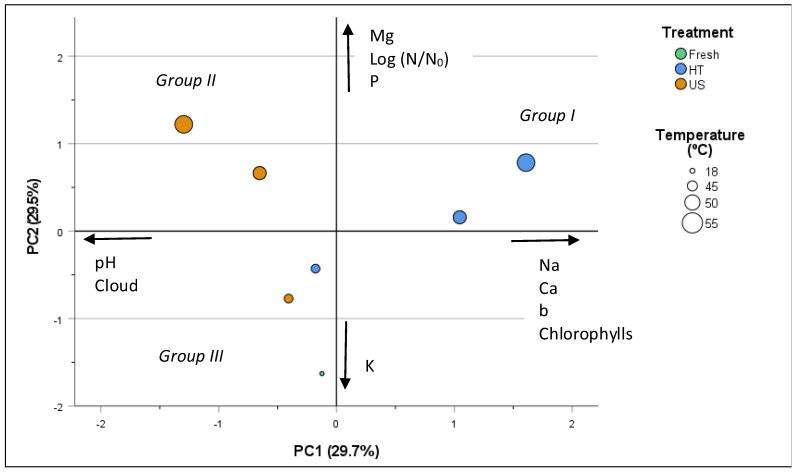
Scores of the two principal components.

A PCA was conducted to minimize the number of experimental variables, thus enhancing the understanding of distinct clusters within the data. Two principal components were assessed, explaining 59% of the total data variance. Variables with loadings higher than 0.6 were considered and labeled on the principal components’ axis (Figure 4).

Curiously, the thermal and ultrasonic treatments at the highest temperatures (50 and 55 °C) fell into separate groups, exhibiting specific distinctive characteristics.

Group I, represented by the heat treatments at temperatures of 50 and 55 °C, is distinguished by its placement on the positive sides of PC1 and PC2, showcasing a correlation with the minerals Na, Ca, Mg, and P, as well as yellowness, higher chlorophyll content, and increased microbial reduction in the samples.

Group II (negative PC1 and positive PC2), comprising the thermosonicated treatments at 50 and 55 °C, is characterized by higher levels of minerals Mg and P, pH closer to that of the fresh juice, elevated cloudiness, and increased microbial reduction loads.

In Group III (negative PC1 and negative PC2), the fresh juice (control) stands out for the highest levels of K, while ultrasonication at the lowest temperature of 45 °C resulted in higher cloudiness compared to the thermal treatment.

## 4. Conclusions

Thermosonication has proven to be a highly effective method for achieving a significant 5-log-cycle reduction in *L. innocua*, meeting FDA requirements for juice processing efficiency. The study found that the required time for a complete inactivation varied with temperature, with 55 °C exhibiting remarkable effectiveness, attaining a 6-log reduction after only 3 min of treatment. In contrast, traditional heat treatments needed longer processing times for comparable reductions in bacterial load.

Despite the efficacy of bacterial inactivation, the study also highlighted the importance of considering the impact on juice quality. Both thermosonication and heat treatments successfully maintained the pH and soluble solid content (SSC) in kiwi juice, with minor alterations observed only in the pH of juice heat-treated at 55 °C. While both treatments affected juice color, thermosonication, particularly at 45 °C, displayed better preservation based on the lowest total color difference (TCD) value. Thermosonication also outperformed heat treatment in enhancing the cloud value and retaining essential nutrients and bioactive compounds in kiwi juice, including total phenolics, chlorophylls, and critical minerals (K, P, Mg, and Ca). The highest temperature in thermosonication stood out for effectively preserving these components.

Therefore, thermosonication emerges as a viable alternative to traditional heat treatments, ensuring *L. innocua* inactivation while preserving nutrients, bioactive compounds, and important physicochemical characteristics. However, additional research on the mechanistic aspects of how thermosonication influences the retention of essential nutrients and bioactive compounds in kiwi juice will contribute to a deeper understanding of the underlying processes. This may lead to the development of targeted interventions to enhance the preservation of specific components during thermosonication. Additionally, future research directions should delve into optimizing thermosonication parameters to strike a balance between bacterial inactivation and the preservation of juice quality in different fruit juices. Inactivation studies with other microorganisms of concern, such as *Alicyclobacillus acidoterrestris*, should also be conducted. Long-term storage studies and sensory evaluations are also required to provide insights into the shelf stability and consumer acceptability of thermosonicated juices.

## Figures and Tables

**Table 1:** Characterization of fresh kiwi juice.

Compound	Content (Mean ± Standard Deviation)
pH	3.62 ± 0.03
SSC (°Brix)	9.93 ± 1.25
Cloud value	0.46 ± 0.32
Color	
L*	22.78 ± 2.76
a*	−2.54 ± 1.49
b*	6.07 ± 2.39
Total phenolics (GAE μg/mL)	0.40 ± 0.32
Total Chlorophylls (μg/mL)	6.23 ± 3.91
Chlorophyll a (μg/mL)	4.06 ± 2.74
Chlorophyll b (μg/mL)	2.17 ± 1.28
Minerals (mg/mL)	
P	1.01 ± 0.46
Mg	0.34 ± 0.08
Ca	0.36 ± 0.08
Na	1.48 ± 4.75
K	18.33 ± 4.68

SSC—Soluble solid content; P—phosphorus; Mg—magnesium; Ca—calcium; Na—sodium; K—potassium.

**Table 2:** Normalized color parameters of thermally treated and thermosonicated kiwi juice.

Treatment	Color Parameters		
	L*	a*	b*
Fresh	1.00 ^a^	1.00 ^ab^	1.00 ^ab^
45 °C	1.03 ± 0.07 ^a^	0.72 ± 0.49 ^a^	0.95 ± 0.20 ^ab^
50 °C	1.06 ± 0.16 ^ab^	0.84 ± 0.35 ^ab^	0.97 ± 0.34 ^ab^
55 °C	1.13 ± 0.03 ^c^	1.16 ± 0.06 ^b^	1.12 ± 0.19 ^b^
45 °C + US	1.12 ± 0.05 ^bc^	1.07 ± 0.40 ^ab^	1.01 ± 0.36 ^ab^
50 °C + US	1.10 ± 0.08 ^bc^	1.03 ± 0.47 ^ab^	0.92 ± 0.33 ^ab^
55 °C + US	1.09 ± 0.04 ^bc^	0.92 ± 0.28 ^ab^	0.80 ± 0.28 ^a^

Values are mean ± confidence intervals at 95% of the three replicates for each treatment. For a given treatment, values with different letters differ significantly (*p* < 0.05).

**Table 3:** Normalized mineral composition of thermally treated and thermosonicated kiwi juice.

Treatment	Elements				
	Ca	P	Mg	Na	K
Fresh	1.00 ^ab^	1.00 ^a^	1.00 ^a^	1.00 ^b^	1.00 ^b^
45 °C	0.92 ± 1.05 ^a^	1.00 ± 0.73 ^a^	1.13 ± 0.11 ^b^	0.99 ± 0.10 ^ab^	0.91 ± 0.05 ^a^
50 °C	1.10 ± 0.34 ^b^	1.18 ± 0.13 ^b^	1.16 ± 0.33 ^b^	1.01 ± 0.05 ^b^	0.94 ± 0.13 ^ab^
55 °C	1.10 ± 0.69 ^b^	1.17 ± 0.69 ^b^	1.16 ± 0.06 ^b^	1.01 ± 0.10 ^b^	0.95 ± 0.39 ^ab^
45 °C + US	0.87 ± 0.31 ^a^	1.03 ± 0.74 ^ab^	1.00 ± 0.14 ^a^	1.00 ± 0.01 ^b^	1.00 ± 0.05 ^b^
50 °C + US	0.94 ± 0.51 ^a^	1.12 ± 0.43 ^ab^	1.15 ± 0.29 ^b^	0.99 ± 0.12 ^ab^	0.92 ± 0.40 ^a^
55 °C + US	0.95 ± 0.06 ^a^	1.12 ± 0.83 ^ab^	1.15 ± 0.16 ^b^	0.97 ± 0.13 ^a^	0.94 ± 0.04 ^ab^

Values are mean ± confidence intervals at 95% of the three replicates for each treatment. For a given mineral, values with different letters differ significantly (*p* < 0.05).

**Table 4:** Weibull model parameters (δ and n) and adjusted coefficient of determination (R^2^_adj_) and root mean squared error (RSME) obtained for the different treatments.

Treatment	δ (min)	n	R^2^_adj_	RSME
45 °C	23.31 ± 3.51	1.64 ± 0.30	0.95	0.38
50 °C	5.06 ± 1.73	0.91 ± 0.25	0.85	0.55
55 °C	2.50 ± 0.70	1.22 ± 0.27	0.95	0.44
45 °C + US	3.19 ± 0.59	1.01 ± 0.14	0.95	0.37
50 °C + US	1.47 ± 0.59	0.94 ± 0.23	0.90	0.64
55 °C + US	0.46 ± 0.21	1.08 ± 0.32	0.90	0.84

Values are mean ± half of the confidence intervals at 95%.

## Data Availability

The original contributions presented in the study are included in the article/supplementary material, further inquiries can be directed to the corresponding author.

## Supplementary Materials

The following supporting information can be downloaded at: https://www.mdpi.com/article/10.3390/foods13020328/s1, Table S1: Chroma and hue angle parameters of thermally treated and thermosonicated kiwi juice.
