# Supplementary material for: Application of Ultrasound Treatments in the Processing and Production of High-Quality and Safe-to-Drink Kiwi Juice
Source: Foods. 2024 Jan 20;13(2):328. doi: 10.3390/foods13020328 (PMC10815408; doi:10.3390/foods13020328)
Supplement: Supplementary file 1 [file foods-13-00328-s001.zip › foods-2791172-supplementary.pdf]

Table S1. Chroma and hue angle parameters of thermally treated and thermosonicated kiwi juice

Treatment	Color parameters	
	**Chroma**	**Hue angle**
Fresh	6.58 ± 0.53 [ab]	112.56 ± 1.30 [ab]
45 °C	6.56 ± 0.69 [ab]	109.39 ± 3.24 [a]
50 °C	6.80 ± 1.02 [ab]	112.11 ± 1.70 [ab]
55 °C	8.05 ± 1.51 [b]	116.04 ± 0.56 [b]
45 °C + US	6.38 ± 1.65 [ab]	113.90 ± 3.02 [ab]
50 °C + US	5.88 ± 1.51 [a]	114.69 ± 4.24 [b]
55 °C + US	4.93 ± 0.86 [a]	117.05 ± 3.68 [b]

Values are mean ± confidence intervals at 95% of the three replicates for each treatment. For a given treatment, values with different letters differ significantly ($p<0.05$).
